# Supplementary material for: Multi‐dataset identification of innovative feature genes and molecular mechanisms in keratoconus
Source: J Cell Mol Med. 2024 Sep 19;28(18):e70079. doi: 10.1111/jcmm.70079 (PMC11412914; doi:10.1111/jcmm.70079)
Supplement: Supplementary file 2 — Material S2. [file JCMM-28-e70079-s002.zip › 7.GSEA analysis/6.SNORA74B/KEGG_HOMOLOGOUS_RECOMBINATION.html]

Details for gene set KEGG\_HOMOLOGOUS\_RECOMBINATION[GSEA]

|  || Dataset | SNORA74B.group.cls#h\_versus\_l |
| Phenotype | group.cls#h\_versus\_l |
| Upregulated in class | h |
| GeneSet | KEGG\_HOMOLOGOUS\_RECOMBINATION |
| Enrichment Score (ES) | 0.27408075 |
| Normalized Enrichment Score (NES) | 0.87310237 |
| Nominal p-value | 0.58979595 |
| FDR q-value | 1.0 |
| FWER p-Value | 1.0 |
Table: GSEA Results Summary

  

Fig 1: Enrichment plot: KEGG\_HOMOLOGOUS\_RECOMBINATION      
 Profile of the Running ES Score & Positions of GeneSet Members on the Rank Ordered List

  

| SYMBOL | TITLE | RANK IN GENE LIST | RANK METRIC SCORE | RUNNING ES | CORE ENRICHMENT || 1 | RPA4 | na | 613 | 0.566 | 0.1683 | Yes |
| 2 | XRCC2 | na | 1591 | 0.408 | 0.2741 | Yes |
| 3 | XRCC3 | na | 10130 | 0.090 | 0.0577 | No |
| 4 | SSBP1 | na | 13992 | 0.036 | -0.0418 | No |
| 5 | POLD4 | na | 14153 | 0.034 | -0.0353 | No |
| 6 | POLD1 | na | 14676 | 0.028 | -0.0411 | No |
| 7 | TOP3A | na | 16192 | 0.013 | -0.0806 | No |
| 8 | RAD50 | na | 18775 | -0.015 | -0.1501 | No |
| 9 | RPA1 | na | 18927 | -0.016 | -0.1492 | No |
| 10 | RAD51B | na | 19629 | -0.024 | -0.1613 | No |
| 11 | TOP3B | na | 20002 | -0.028 | -0.1627 | No |
| 12 | RAD51C | na | 20278 | -0.031 | -0.1603 | No |
| 13 | MUS81 | na | 21999 | -0.052 | -0.1928 | No |
| 14 | RPA2 | na | 22266 | -0.055 | -0.1824 | No |
| 15 | POLD2 | na | 22465 | -0.058 | -0.1692 | No |
| 16 | BLM | na | 23507 | -0.073 | -0.1752 | No |
| 17 | POLD3 | na | 23900 | -0.079 | -0.1605 | No |
| 18 | RAD54B | na | 24322 | -0.086 | -0.1443 | No |
| 19 | RPA3 | na | 25111 | -0.100 | -0.1340 | No |
| 20 | RAD52 | na | 25358 | -0.104 | -0.1068 | No |
| 21 | RAD54L | na | 25472 | -0.107 | -0.0749 | No |
| 22 | BRCA2 | na | 26233 | -0.122 | -0.0569 | No |
| 23 | NBN | na | 29127 | -0.198 | -0.0750 | No |
| 24 | EME1 | na | 29225 | -0.201 | -0.0117 | No |
| 25 | RAD51D | na | 29856 | -0.220 | 0.0425 | No |
| 26 | RAD51 | na | 31546 | -0.298 | 0.0917 | No |
Table: GSEA details [plain text format]

  

Fig 2: KEGG\_HOMOLOGOUS\_RECOMBINATION      
 Blue-Pink O' Gram in the Space of the Analyzed GeneSet

  

Fig 3: KEGG\_HOMOLOGOUS\_RECOMBINATION: Random ES distribution      
 Gene set null distribution of ES for **KEGG\_HOMOLOGOUS\_RECOMBINATION**
